# Reduced left dorsolateral prefrontal activation in problematic smartphone users during the Stroop task: An fNIRS study

**DOI:** 10.3389/fpsyt.2022.1097375

**Published:** 2023-01-09

**Authors:** Ming-Qiang Xiang, Long- Lin, Yun-Ting Song, Min Hu, Xiao-Hui Hou

**Affiliations:** ^1^School of Sport and Health, Guangzhou Sport University, Guangzhou, China; ^2^Guangdong Key Lab of Physical Activity and Health Promotion, Guangzhou Sport University, Guangzhou, China; ^3^Scientific Research Center, Guangzhou Sport University, Guangzhou, China

**Keywords:** problematic smartphone use, inhibitory control, fNIRS, Stroop interference, prefrontal cortex

## Abstract

**Introduction:**

The widespread use of smartphones has triggered concern over problematic smartphone use (PSPU), as well as the need to elucidate its underlying mechanisms. However, the correlation between cortical activation and deficient inhibitory control in PSPU remains unclear.

**Methods:**

This study examined inhibitory control using the color–word matching Stroop task and its cortical-activation responses using functional near-infrared spectroscopy (fNIRS) in college students with PSPU (*n* = 56) compared with a control group (*n* = 54).

**Results:**

At the behavioral level, Stroop interference, coupled with reaction time, was significantly greater in the PSPU group than in the control group. Changes in oxygenated hemoglobin (Oxy-Hb) signals associated with Stroop interference were significantly increased in the left ventrolateral prefrontal cortex, left frontopolar area, and bilateral dorsolateral prefrontal cortex (DLPFC). Moreover, the PSPU group had lower Oxy-Hb signal changes associated with Stroop interference in the left-DLPFC, relative to controls.

**Discussion:**

These results provide first behavioral and neuroscientific evidence using event-related fNIRS method, to our knowledge, that college students with PSPU may have a deficit in inhibitory control associated with lower cortical activation in the left-DLPFC.

## Introduction

Smartphones are popular devices that put the world at our fingertips and bring convenience to our increasingly digital lifestyles. However, excessive smartphone use and its potential negative consequence have drawn increasing attention (1, 2). Problematic smartphone use (PSPU) has been defined as excessive, problematic, psychosocially dysfunctional smartphone use that can be considered a behavioral addiction (3, 4). Several psychometric instruments have been developed to measure PSPU, including the Problematic Mobile Phone Use Questionnaire (PMPU-Q) (5) and the Smartphone Addiction Scale (SAS) (6), as well as its short version (SAS-SV) (3). Using such instruments, high prevalence rates of PSPU, ranging from 12.5 to 33.9%, have been identified, especially among students (7–9). Considering its high prevalence and potentially harmful effects, it is important to explore the underlying mechanisms of PSPU, especially its neuropsychological mechanisms (10, 11). Such work could help to further our understanding of PSPU behaviors, identify potential smartphone addicts, and develop targeted interventions.

Prior research has provided evidence that the prominent feature of behavioral addiction is deficient inhibitory control (12, 13), which manifests as an inability to volitionally suppress prepotent responses. Recent behavioral data suggests that inhibitory control deficit might be intrinsically linked to PSPU, individuals with higher levels of PSPU tend to have poorer inhibitory control, and increased inhibitory control is associated with a lower level of PSPU (14–16). Evidence from neuroimaging studies suggests that PSPU might also show such characteristics. For example, using functional magnetic resonance imaging (fMRI), Horvath et al. (10) found that individuals with PSPU had significantly lower anterior cingulate cortex (ACC) activity than the control (14) group. In addition, a smaller gray-matter volume in the orbitofrontal cortex (OFC) was found in individuals with PSPU compared to controls (17). Such ACC and OFC abnormalities have been identified as related to increased craving and decreased inhibitory control (18, 19). Thus, the abovementioned fMRI studies revealed that alterations in brain structure and functioning were implicated in lower inhibitory control in PSPU. Moreover, event-related potential (ERP) has been used to search for the neural substrates of inhibitory control in PSPU. Specifically, the N2 and P3 components are believed to be related to inhibitory control in the early or late stage of the inhibitory process (20, 21). A number of studies have found a weaker P3 amplitude or larger N2 amplitude in PSPU groups compared to control groups during inhibitory control tasks (22, 23). This suggests that individuals with PSPU might have general deficits in inhibitory control.

Event-related potential provides essential temporal information relating to inhibitory control task-induced brain activities, although only rough information on its origin in the brain is revealed. To examine the specific brain regions that undergo changes in activation in response to inhibitory effects in people with PSPU, this investigation employed functional near-infrared spectroscopy (fNIRS). The non-invasive optical method fNIRS can detect hemodynamic alterations in the cortex by quantifying changes in near-infrared light that pass through the skull (24, 25). This technique has been proven to be effective in assessing cortical-activity responses to alterations in oxygenation based on the association of neuronal activity to regional cerebral blood flow (26, 27). Unlike other neuroimaging techniques, including fMRI, fNIRS has many advantages, which comprise high temporal resolution, low cost, and tolerance of motion artifacts, and relative portability (28, 29). It has been widely applied in cognitive and social neuroscience research (30). However, to our knowledge, fNIRS has not been employed to investigate the correlation between cortical-activation and inhibitory control in individuals diagnosed with PSPU.

Stroop color-word matching pertains to a classical approach of measuring prefrontal cortex (PFC) function (31) and has been widely utilized in neuroimaging studies such as fNIRS (32, 33), as well as regions of brain activity that are related to the task. Pearlier neuroimaging studies such as fMRI have revealed a correlation between the cognitive conflict in relation to the Stroop paradigm and the ACC and the lateral PFC (LPFC), particularly the dorsolateral PFC (DLPFC), in which the ACC is influenced by conflict; in addition, the DLPFC is thought to implement cognitive control (34, 35). Because fNIRS is limited to assessing lateral cortical surfaces, it is incapable of monitoring cortical activation in the ACC, although it has been successfully utilized in investigating Stroop interference-related cortical activation within the LPFC (36). Therefore, the present study selected the PFC as our region of interest (ROI) and we analyzed the LPFC with fNIRS probes that cover the region.

In summary, we investigated inhibition control in the PSPU with the Stroop-task paradigm to evaluate behavioral and cortical-activation responses. With an event-related multichannel fNIRS that targeted the LPFC, we characterized the cortical-activation pattern in the color-word matching Stroop task in an PSPU group in relation to a control group. The hypotheses of this study were that compared to controls, individuals with PSPU possess inhibitory control deficiency at the behavioral level, as well as that is associated with reduced cortical activation in specific LPFC regions.

## Materials and methods

### Participants

Participants were recruited through the use of flyers posted at a university campus and by ads on social media platforms such as WeChat Moments. A total of 347 college students expressed interest in the study *via* phone or WeChat; 298 provided demographic information and filled out the self-control scale (37) and the SAS-SV (3), and took screenshots of their iPhone (iOS STT) or Android (e.g., Healthy Cell Phone Use in Huawei) screen time and upload their smartphone screen time for the last 7 days. We assessed the severity of smartphone dependency using the self-reported SAS-SV. The scale has been translated into Chinese version with good reliability and validity (9), and the coefficient alpha of this study was 0.862. Participants were assigned to a high-risk group when SAS-SV scores exceeded cutoff points of >31 for males and >33 for females (3).

The following eligibility criteria were used: (1) right-handed smartphone owners within the age range of 18–25 years, (2) normal or corrected-to-normal vision in the absence of color blindness, (3) no general contraindications to fNIRS, and (4) no self-reported history of mental illness or neurological disease.

The participants were invited to the clinician-administered interview, in order to assess smartphone usage patterns, SAS-SV scores (high-risk or low-risk), and their level of smartphone dependency using diagnostic criteria (e.g., preoccupation, tolerance, withdrawal, persistence, escape, problems, deception, displacement, and conflict), for diagnosing Internet gaming disorder which has been described in the (see section “3 Results”) of the 2013 Diagnostic and Statistical Manual of Mental Disorders, 5th edition (DSM-5). Based on the clinician-administered interview, we identified 58 participants with PSPU from the 298 participants in the sample. Fifty-eight participants from the remaining non-PSPU participants were assigned to the control group. During the experiment, two subjects voluntarily dropped out and four were excluded due to technical failure, resulting in a total of 56 subjects in the PSPU group and 54 in the control group.

This study received approval from the Human Experimental Ethics Board of the respective authors’ university and was conducted in compliance with the Declaration of Helsinki. All subjects agreed to participate in the fNIRS experiment and signed an informed consent form. All participants received monetary compensation (RMB 50 yuan) after completing the experiment.

### Behavioral measurements

The color-word matching Stroop test was utilized to assess inhibitory control involving an event-related design (31, 32). Stimuli included three color words, namely, RED, GREEN, and BLUE that were presented in Chinese as 红, 绿, and 蓝, respectively. The font size of the Chinese characters was 2 cm^2^, and each character was positioned at the center of a 21-inch screen by E-prime 2.0 software. The Stroop task comprised 30 trials, which included 15 congruent and 15 incongruent trials, which were randomly introduced. In the congruent trials, words and colors matched (i.e., RED appeared in red), whereas in the incongruent condition, words and colors were mismatched (i.e., RED appeared in blue). The participants were asked to identify the color of the presented word by pressing a corresponding button on the standard computer keyboard as fast as they could with minimal error. We put the red, green and blue paper labels on the keyboard “J, K, and L,” respectively. Participants were instructed to use right index finger for red, right middle finger for green, and right ring finger for blue. Participants were given a practice session to familiarize themselves with the mappings.

Each trial started with a red fixation cross that appeared in the center of a black screen for 500 ms and followed by a blank interval for either 300 ms or 500 ms (randomly selected) to prevent prediction of the timing of word stimuli (38). The target word was then shown on the screen for 200 ms, which was followed by a blank interval of 2,000 ms or upon generation of a response. Each trial was independently presented with an interstimulus interval using a white fixation cross for 12 s. We performed eight practice trials before launching the experiment, and participants started to do the formal experiment until they have got 100% accuracy. Response times and accuracy rates were recorded.

### fNIRS measurements

**FIGURE 1:**
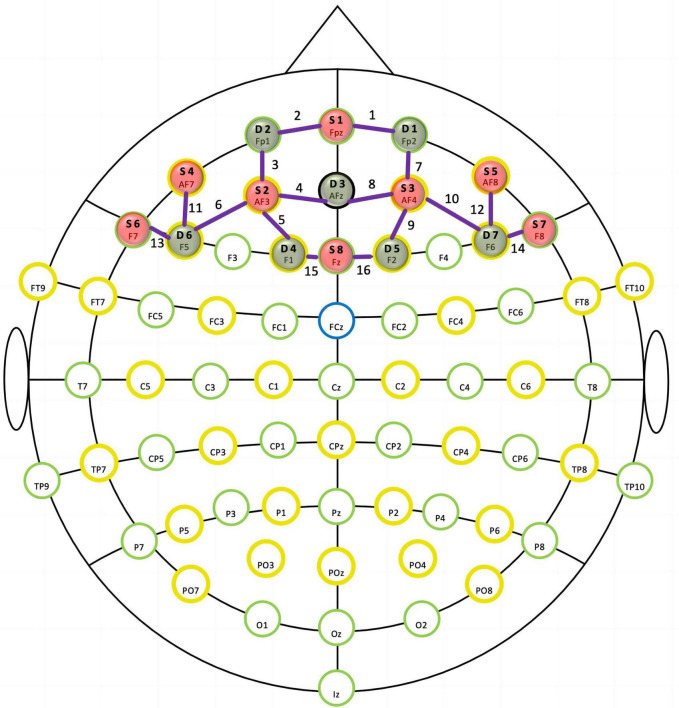
In the functional near-infrared spectroscopy (fNIRS) montage, sources (S) and detectors (D) were situated according to the international 10–20 system. The purple lines and numbers represent channels.

A multichannel fNIRS optical topography system (NIRSport, NIRx Medical Technologies, LLC, New York, NY, USA) was used to monitor cortical hemodynamic changes in the PFC during the Stroop task. The machine emits near-infrared lights at two wavelengths (760 and 850 nm). Signals representing changes in oxygenated hemoglobin (Oxy-Hb) and deoxygenated hemoglobin (Deoxy-Hb) levels were calculated using a modified Beer–Lambert law (39). Our montage setup comprised eight light sources and eight detectors that were alternately arranged at interprobe distances of approximately 3 cm, resulting in 16 channels (Ch). We positioned the NIRScap on the head by centering the probe bottom row to the Fpz position. We used an fNIRS Optodes’ Location Decider (fOLD) with the maximum probability method to define the locations of the NIRS channels (40). We recorded hemodynamic responses in lateral prefrontal activation, including six ROIs—namely, the left and right dorsolateral PFC (l-DLPFC, r-DLPFC), left and right ventrolateral PFC (l-VLPFC, r-VLPFC), and left and right frontopolar area (l-FPA, r-FPA). Figure 1 shows an overview of the spatial organization of the fNIRS optode placement, and the corresponding ROIs can be found in the Supplement (Supplementary Table 1).

### fNIRS data analysis

We used NIRStar acquisition software (NIRx Medical Technologies, LLC, New York, NY, USA) in recording fNIRS data and in evaluating the signal-to-noise ratio. fNIRS data were preprocessed with the nirsLAB analysis package (v2017.06, NIRx Medical Technologies, LLC, Los Angeles, CA, USA). Detection and removal of spike artifacts and faulty channels were performed in subsequent analyses. Later, we applied a band filter with a 0.01-Hz low-cutoff frequency to discard baseline drift and a 0.1-Hz high-cutoff frequency to decrease respiratory noise, including heartbeat pulsations, skin blood flow, and blood pressure (41). We employed the modified Beer–Lambert law to assess hemoglobin concentration changes (39). For primary metrics, we evaluated concentrations of Oxy-Hb and Deoxy-Hb using a 7.81-Hz sampling rate.

### Statistical analyses

Statistical analyses were conducted using the SPSS Statistical Package (SPSS, Inc., Chicago, IL, USA). We assessed accuracy (AC) and reaction time (RT) by repeated-measures two-way ANOVA, using task (incongruent/congruent) as within-subject factors, whereas group (control/PSPU) as between-subject factors, to evaluate the reproducibility of the Stroop task at all conditions. Subsequently, the [incongruent–congruent] contrast for AC or RT, which represented Stroop interference, was determined to identify differences between the PSPU and control groups with an independent-sample *t*-test. In addition, we also used the [incongruent–congruent] contrasts for Oxy-Hb or Deoxy-Hb levels to investigate cortical regions that responded to Stroop interference. The average of the [incongruent–congruent] contrasts of the PSPU and control groups were used as substrates for ROI-wise analysis alongside a false discovery rate (FDR) control.

## Results

### Demographics and psychometric assessment

In terms of age and gender, there was no significant difference between PSPU and control groups. Comparing to controls, individuals with PSPU significantly spent more time using smartphones, having lower scores in the SAS and self-control scales (see Table 1).

**TABLE 1:** Participant demographics and psychometric scores for individuals with problematic smartphone use (PSPU) and control groups.

	PSPU (*n* = 56)	Control (*n* = 54)	*t/*χ^2^	*p*
	**Mean ± SD**	**Mean ± SD**		
Age (years)	20.46 ± 1.36	20.52 ± 1.03	0.244	0.807
Gender (m/f)	34/22	23/31	3.616※	0.057
Time on smartphone use (min/day)	508.36 ± 97.86	275.67 ± 104.07	12.085	<0.001
SAS-SV total score	38.50 ± 4.77	27.91 ± 4.28	12.248	<0.001
Self-control score	58.25 ± 7.94	65.11 ± 9.97	4.000	<0.001

SAS-SV, short version of the Smartphone Addiction Scale.※indicates χ^2^ test.

### Behavioral results

**FIGURE 2:**
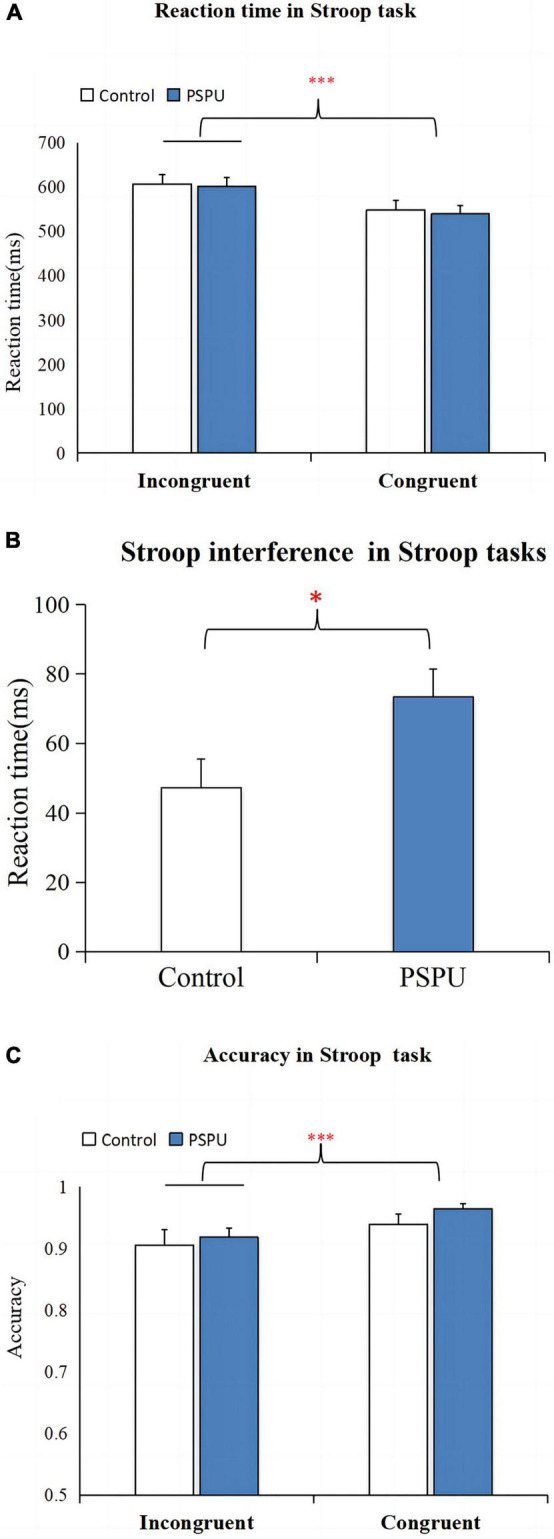
Stroop task performance. **(A)** Comparison of RT between congruent and incongruent task. **(B)** Comparison of Stroop interference on RT between the control and PSPU groups. **(C)** Comparison of AC between congruent and incongruent task. All data are expressed as the mean ± standard error. ^***^p ≤ 0.001.

Table 2 depicts the AC and RT for both groups. We conducted a two-way ANOVA to examine whether group (PSPU vs. controls) and task (incongruent vs. congruent) affected the recognition RT. We found a significant main effect of task [*F*(1,108) = 194.352, *p* < 0.001, partial η^2^ = 0.643] (Figure 2A), suggesting a Stroop interference effect between congruent and incongruent tasks. However, we did not find a main effect of group (*p* > 0.05). There was also a significant interaction between task and group [*F*(1,108) = 10.751, *p* = 0.001, partial η^2^ = 0.091]. Simple effect analyses revealed RT for incongruent task were significantly greater than for the congruent task in both group (*p* < 0.001). However, the *t*-test analyses showed that Stroop interference was significantly higher in individual with PSPU relative to the control group (*t* = 3.283, *p* = 0.001) (Figure 2B). We also conducted the analysis on AC, we found a significant main effect task [*F*(1,108) = 24.192, *p* < 0.001, partial η^2^ = 0.183] (Figure 2C). There is no main effect of group, nor interaction between task and group, the following analyses was collapsed.

**TABLE 2:** Accuracy (AC) and reaction time (RT) for individuals with problematic smartphone use (PSPU) and control groups.

	PSPU (*n* = 56)	Control (*n* = 54)	*t*	*p*
	**Mean ± SD**	**Mean ± SD**		
**Accuracy (AC)**				
Incongruent	0.918 ± 0.109	0.905 ± 0.187	0.443	0.659
Congruent	0.965 ± 0.064	0.939 ± 0.122	1.362	0.176
Stroop interference	0.046 ± 0.078	0.034 ± 0.093	0.733	0.465
**Reaction time (RT)**				
Incongruent	667.512 ± 153.721	609.673 ± 154.291	1.969	0.051
Congruent	564.226 ± 144.711	545.720 ± 156.929	0.643	0.521
Stroop interference	103.285 ± 65.066	63.954 ± 60.567	3.283	0.001

### fNIRS results

**FIGURE 3:**
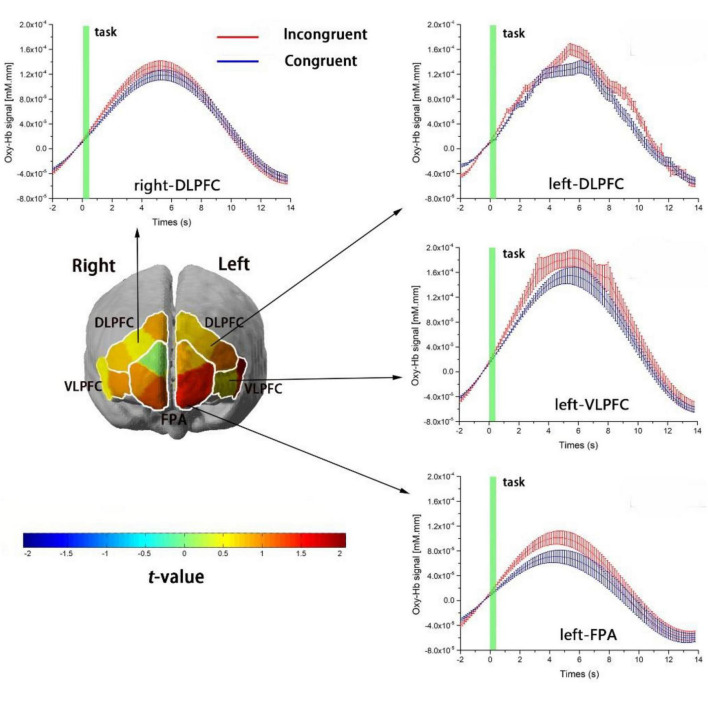
Cortical-activation patterns involving the Stroop task. The four graphs show the timelines for Oxy-Hb signals as responses to the incongruent (red) and congruent (blue) tasks in the left DLPFC, right DLPFC, left VLPFC, and left FPA. The timelines are adjusted to the average value at baseline (i.e., 2 s before task onset). Changes in Oxy-Hb signals are presented in arbitrary units (mM mm). Error bars represent the standard error at various time points. The left figure is a t-map of changes in the Oxy-Hb signal that reflect the effect of Stroop interference (incongruent–congruent); *t*-values are presented using the color bar.

We conducted paired-sample *t*-test on each ROI to investigate the cortical regions in response to Stroop interference between congruent and incongruent tasks in both group. The results showed that cortical hemodynamic changes in Oxy-Hb associated with Stroop interference (incongruent vs. congruent) were significant in four of the six ROIs: left-DLPFC, left -VLPFC, left-FPA, and right-DLPFC (paired-sample *t*-test, *p* < 0.05, FDR controlled). Figure 3 shows cortical-activation patterns in response to the congruent and incongruent tasks with each ROI’s timeline of Oxy-Hb. However, there was no statistical significance for Deoxy-Hb changes in all ROIs in response to Stroop interference.

**FIGURE 4:**
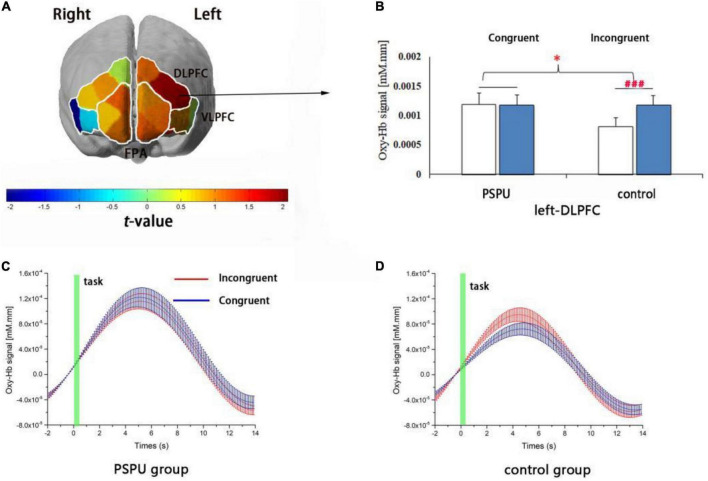
Cortical activation in relation to the effect of Stroop interference. **(A)** t-map of changes in Oxy-Hb signals that reflect the effect of Stroop interference. Significant differences between the PSPU and control groups are observed in the left-DLPFC (*p* = 0.02, FDR controlled) using the six regions of interest. **(B)** Changes in individual Stroop task-related Oxy-Hb levels in the left-DLPFC. Oxy-Hb hemodynamic alterations due to incongruent tasks are significantly higher than congruent tasks only in the control group (*p* < 0.001). **(C,D)** depict the timelines of Oxy-Hb changes in their response to congruent and incongruent tasks within the left-DLPFC involving the PSPU and control groups, respectively.

The ANOVAs for the six ROIs revealed a main effect of task in the left-DLPFC [*F*(1,108) = 11.86, *p* = 0.004, partial η^2^ = 0.074] and a significant interaction between group (control/PSPU) and task (incongruent/congruent) factors in the left-DLPFC [*F*(1,108) = 10.125, *p* = 0.002, partial η^2^ = 0.086]. However, no main effect of group was observed (*p* > 0.05). Simple effect analyses revealed Oxy-Hb hemodynamic changes related to the incongruent task in the left-DLPFC were significantly greater than for the congruent task in the control group [*F*(1,108) = 18.354, *p* < 0.001, partial η^2^ = 0.145], while there were no significant differences in the PSPU group. Moreover, Oxy-Hb hemodynamic changes related to Stroop interference in the left-DLPFC were significantly lower in the PSPU group than in the control group (*p* = 0.02, FDR controlled; see Figure 4). Supplementary Table 2 summarizes Oxy-Hb hemodynamic changes related to Stroop interference in all ROIs for the control and PSPU groups. However, we did not observe any significant effects of interactions and main effects of Deoxy-Hb in all ROIs.

## Discussion

This study used fNIRS to investigate inhibition control in college students with PSPU. The results revealed that, relative to the control group, college students with PSPU had higher RT and lower cortical activation in the left DLPFC related to Stroop interference, indicating that those with PSPU had deficits in inhibitory control at the behavioral and neural level. Therefore, our hypothesis was supported.

At the behavioral level, Stroop interference with RT was significantly higher in the PSPU group than in the control group, suggesting that individuals with PSPU had deficient inhibitory control. This result is contradictory to previously reported findings using go/no-go tasks, which showed that there is no general deficient inhibitory control in the PSPU group (23, 42). The reason might be that such Stroop task was more sensitive than go/no-go task for examining deficient inhibitory control in behavioral levels. However, Gao et al. (22) using a mobile app-background go/no-go task, found that individuals with PSPU had more commission errors than those in the control group. These mobile phone cues may trigger impulsive behavior as well as failure of inhibitory control in particular situations. This suggests that a mobile phone cue task might be a sensitive measure for examining deficient inhibitory control in behavioral levels among individuals with PSPU.

To elucidate the underlying neural mechanism, we employed an event-related fNIRS method to assess changes in oxy-hemodynamic response, mainly focusing on the Stroop-interference effect. We detected significant increases in Oxy-Hb signal changes that were associated with Stroop interference within the bilateral DLPFC, left VLPFC, and left FPA. This specific neural basis for Stroop interference is concordant to the findings of previous fNIRS studies (26, 43), as well as with other fMRI neuroimaging studies that have reported that the LPFC, in conjunction with ACC, is an essential brain region that reflects interference processing/response inhibition (44, 45). However, with the limited depth penetration of lights in fNIRS, we limited our functional arguments to the LPFC.

Upon confirmation of a reliable neural substrate for the Stroop-interference effect, we showed that the PSPU group exhibited lower Stroop interference-related cortical activation within the left-DLPFC relative to the control group. Our findings support the view that individuals with PSPU have an inhibitory function defect at the neural level. These results are congruent with previous ERP studies using go/no-go tasks. For example, Chen et al. (23) reported a more negative N2 ERP in excessive smartphone users relative to normal users, indicating a deficit in inhibition processing at the early stages. Similar findings have been reported for those who excessively use social networking sites, showing an especially larger N2 amplitude and smaller no-go P3 amplitude when participants were confronted with images related to such sites (42). The results of this fNIRS study and those of previous ERP studies imply that individuals with PSPU may experience more conflicts and exhibit a general deficit in inhibition-control processing.

A novelty of this study is that we provide first behavioral and neuroscientific evidence using event-related fNIRS method, to our knowledge, that college students with PSPU may have a deficit in inhibitory control associated with lower cortical activation in the left-DLPFC. Recent fNIRS studies have investigated the contribution of DLPFC to various cognitive processes, including dual-motor and cognitive-task performance (46), word encoding involving a memory task (47), as well as mental effort (48). Furthermore, the DLPFC and parietal cortex constitute the frontoparietal network, which plays a critical role in goal-directed behavior and top-down control (49). Dual-system theories have suggested that human behavior is guided by reflexive (automatic) and reflective (control) systems (50). Several studies have found that individuals with PSPU who had deficient inhibitory control are more likely to immediately respond to mobile phone messages (11, 22). Combining our results with previous findings, we can suggest that a deficit in inhibition control in individuals with PSPU might be the result of reduced top-down processes (e.g., self-regulation) and strong bottom-up ones (e.g., mobile phone cues). However, this interpretation needs to be assessed by the structural and functional connectivity of the brain networks in the PSPU group (51, 52). Future research could apply a connectome-based predictive modeling approach to identify the functional connectome supporting deficit in inhibitory control or explore the patterns of a cortical gyrification covariance network associated with PSPU (53, 54).

This study has a number of limitations. First, despite widespread concern on the negative effect of excessive smartphone use on inhibitory control, the extent of smartphone addiction remains unclear (55). Our data for smartphone use behavior did not clearly differentiate between social communication, mobile gaming, and Internet surfing. In addition, although inclusion criteria included no self-reported history of psychiatric or neurological disorders, we did not use relevant scales to specifically assess participants’ levels of anxiety and depression. Patients with anxiety or depression might impair inhibitory control (56, 57). Therefore, the potential variables that influence excessive smartphone use and inhibitory control. Second, the use of fNIRS is limited to monitoring cortical surface activity and cannot be used to monitor deeper regions such as subcortical structures or the ACC. The contribution of these regions to inhibitory control has been reliably evaluated using fMRI in individuals diagnosed with PSPU (10, 58). Future research could explore PSPU brain networks in the context of psychoradiology, a new field at the intersection of psychiatry, psychology and radiology (59). Finally, since we used a cross-sectional rather than longitudinal design, causality between PSPU and inhibitory control remains to be clarified. Future research should use longitudinal designs, experimental manipulation, and more sophisticated statistical methods to clarify the causal relationships between PSPU and inhibitory control at the behavioral and neural levels. Such work could provide more definitive interpretations of our findings.

## Conclusion

The current results demonstrate that college students with PSPU had higher RT and lower cortical activation in the left DLPFC related to Stroop interference, relative to the control group. This suggests that college students with PSPU may have a deficit in inhibitory control associated with lower cortical activation in the left-DLPFC.

## Data availability statement

The original contributions presented in this study are included in the article/Supplementary material, further inquiries can be directed to the corresponding authors.

## Ethics statement

The studies involving human participants were reviewed and approved by the Human Experimental Ethics Board of the Guangzhou Sport University. The patients/participants provided their written informed consent to participate in this study. Written informed consent was obtained from the individual(s) for the publication of any potentially identifiable images or data included in this article.

## Author contributions

MH, X-HH, and M-QX designed the study and wrote the protocols. X-HH and M-QX designed Stroop task and selected the scales. L-L and Y-TS participated in the fNIRS data collection and undertook the statistical analysis. L-L and M-QX drafted the manuscript. All authors contributed to manuscript revision, read, and approved the submitted version.
