# Supplementary material for: Reduced left dorsolateral prefrontal activation in problematic smartphone users during the Stroop task: An fNIRS study
Source: Front Psychiatry. 2023 Jan 9;13:1097375. doi: 10.3389/fpsyt.2022.1097375 (PMC9868828; doi:10.3389/fpsyt.2022.1097375)
Supplement: Supplementary file 1 [file Table_1.DOCX]

**Table S1 General features of all channels (i.e., source-detector combinations) and their corresponding positions based on the international 10–20 system and respective ROIs**

| **ROI** | **Channel** | **Source-detector**  **number** | **Positions corresponding to**  **10–20 system** |
| --- | --- | --- | --- |
| Left-FPA | CH2 | S1-D2 | FPZ-FP1 |
|  | CH3 | S2-D2 | AF3-FP1 |
|  | CH4 | S2-D3 | AF3-AFZ |
| Left-DLPFC | CH5 | S2-D4 | AF3-F1 |
|  | CH6 | S2-D6 | AF3-F5 |
|  | CH15 | S8-D4 | FZ-F1 |
| Left-VLPFC | CH11 | S4-D6 | AF7-F5 |
|  | CH13 | S6-D6 | F7-F5 |
| Right-FPA | CH1 | S1-D1 | FPZ-FP2 |
|  | CH7 | S3-D1 | AF4-FP2 |
|  | CH8 | S3-D3 | AF4-AFZ |
| Right-DLPFC | CH9 | S3-D5 | AF4-F2 |
|  | CH10 | S3-D7 | AF4-F6 |
|  | CH16 | S8-D5 | FZ-F2 |
| Right-VLPFC | CH12 | S5-D7 | AF8-F6 |
|  | CH14 | S7-D7 | F8-F6 |

Note. ROI: region of interest; S: source; D: detector; FPA: frontopolar area; DLPFC: dorsolateral prefrontal cortex; VLPFC: ventrolateral prefrontal cortex.

**Table S2 Stroop-interference-related Oxy-Hb changes in all ROIs (mM**•**mm).**

| ROI | PSPU (n = 56)  Mean ± SE | Control (n = 54) Mean ± SE | ***P***  **(**FDR-corrected**)** |
| --- | --- | --- | --- |
| Left-FPA | 0.0110 ± 0.0100 | 0.0556 ± 0.0193 | 0.13 |
| Left-DLPFC | −0.0015 ± 0.0081 | 0.0358 ± 0.0084* | 0.02 |
| Left-VLPFC | 0.0136 ± 0.0161 | 0.0451 ± 0.0140 | 0.22 |
| Right-FPA | −0.0038 ± 0.0112 | 0.0878 ± 0.0684 | 0.22 |
| Right-DLPFC | 0.0062 ± 0.0081 | 0.0228 ± 0.0081 | 0.22 |
| Right-VLPFC | 0.0464 ± 0.0250 | 0.0084 ± 0.0173 | 0.22 |

Note. SE: standard error; ROI: region of interest, FPA: frontopolar area; DLPFC: dorsolateral prefrontal cortex; VLPFC: ventrolateral prefrontal cortex; * (PSPU vs. control, FDR-corrected, *p* < 0.05).
